# Functional Characterization of a Dihydroflavanol 4-Reductase from the Fiber of Upland Cotton (*Gossypium hirsutum*)

**DOI:** 10.3390/molecules21020032

**Published:** 2016-01-26

**Authors:** Le Wang, Yue Zhu, Peng Wang, Qiang Fan, Yao Wu, Qing-Zhong Peng, Gui-Xian Xia, Jia-He Wu

**Affiliations:** 1 The State Key Laboratory of Plant Genomics, Institute of Microbiology, Chinese Academy of Sciences, Beijing 100101, China; wangle510@foxmail.com (L.W.); imzhuyue@foxmail.com (Y.Z.); wangpeng27085@163.com (P.W.); fanqiang1022@foxmail.com (Q.F.); wuyao@im.ac.cn (Y.W.); 2 Hunan Provincial Key Laboratory of Plant Resources Conservation and Utilization, College of Biology and Environmental Sciences, Jishou University, No. 120 Ren Min South Road, Jishou 416000, Hunan, China; qzpengjsu@163.com

**Keywords:** upland cotton (*Gossypium hirsutum*), dihydroflavanol-4-reductase (DFR), anthocyanins, proanthocyanidins (PAs), flavan-3-ols

## Abstract

Dihydroflavanol 4-reductase (DFR) is a key later enzyme involved in two polyphenols’ (anthocyanins and proanthocyanidins (PAs)) biosynthesis, however it is not characterized in cotton yet. In present reports, a *DFR* cDNA homolog (designated as *GhDFR1*) was cloned from developing fibers of upland cotton. Silencing *GhDFR1* in cotton by virus-induced gene silencing led to significant decrease in accumulation of anthocyanins and PAs. More interestingly, based on LC-MS analysis, two PA monomers, (–)-epicatachin and (–)-epigallocatachin, remarkably decreased in content in fibers of *GhDFR1-*silenced plants, but two new monomers, (–)-catachin and (–)-gallocatachin were present compared to the control plants infected with empty vector. The ectopic expression of *GhDFR1* in an *Arabidopsis TT3* mutant allowed for reconstruction of PAs biosynthesis pathway and led to accumulation of PAs in seed coat. Taken together, these data demonstrate that GhDFR1 contributes to the biosynthesis of anthocyanins and PAs in cotton.

## 1. Introduction

**Figure 1:**
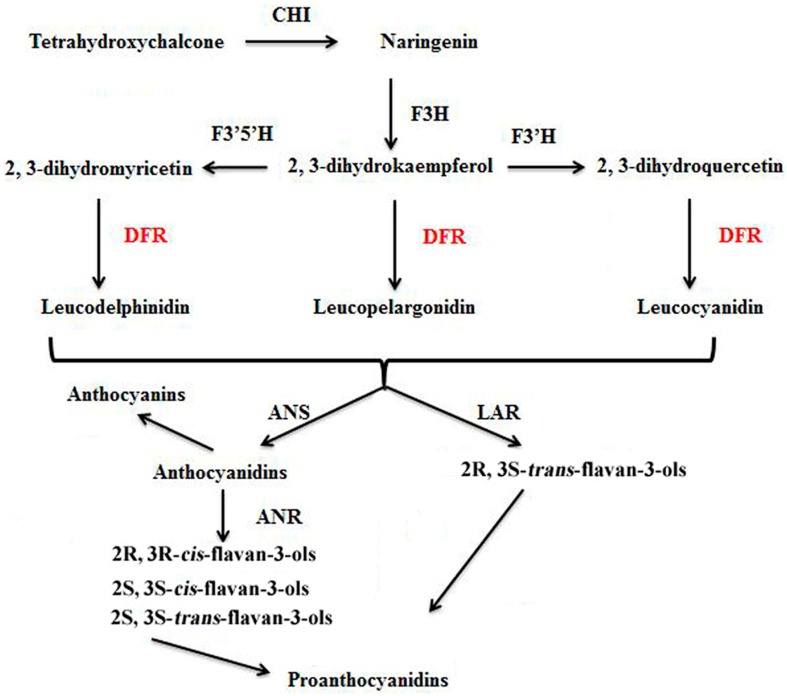
Biosynthetic relationship of DFR to anthocyanins, flavan-3-ols, and proanthocyanidins. CHI: chalcone isomerase; F3H: (2S)-flavanone 3-hydroxylase; F3′H: flavonoid 3′-hydroxylase; F3′,5′H: flavonoid 3′,5′-hydroxylase; DFR: dihydroflavonolreductase; ANS: anthocyanidin synthase (also known as leucoanthocyanidin dioxygenase); LAR: leucoanthocyanidin reductase; ANR: anthocyanidin reductase.

Dihydroflavanol 4-reductase (DFR, EC 1.1.1.219) is a NADPH-dependent oxidoreductase that is a key later enzyme involved in controlling flux into biosynthesis of anthocyanins and proanthocyanidins (PAs), which are two polyphenols [1]. DFR in plants catalyzes the transformation of three dihydroflavonols (dihydrokaempferol, dihydroqeretin, and dihydromyricetin) into the corresponding leucoanthocyanidins (leucopelargonidin, leucocyanidin, and leucodelphinidin), which are ultimately converted into anthocyanins or PAs (Figure 1) [2,3,4]. However, DFRs from some species like *Petunia hybrida* lack the ability to accept dihydrokaempferol as a substrate, so they cannot produce the pelargonidin-based anthocyanins and PAs [2]. To date, many studies have reported the biochemical and genetic characterization of DFR [1,5,6,7,8,9]. However, most of studies focused on the flower and fruit coloration involved in anthocyanin biosynthesis. Some evidences showed DFR in some plants can biosynthesize and accumulate PAs, which is possibly involved in increased resistance/tolerance to pathogens, insects, and herbivores [10].

PAs, also known as condensed tannins (polyphenols), are oligomers or polymers of flavan-3-ols [11], widely producing in land plants such as ferns, gymnosperms, and angiosperms [12]. PAs are mainly found distributed in leaves, stems, fruits, roots and seed coats [12,13,14,15,16]. In general, PAs play multiple protective roles against pathogens, herbivores, and UV damage in plants [11,17,18]. Many studies further demonstrate that the structures and contents of PAs are associated with plants’ resistance/tolerance to pathogens, insects, and herbivores [17,19,20,21]. In addition, anthocyanins are a large group of flavonoid (polyphenols) that are ubiquitously present in plants, and provide red, blue, and purple flowers as well as colorful vascular tissues and fruits [22]. In general, anthocyanins help plants attract insects and animals for pollination and dissemination of seeds, as well as protecting them from UV-damage [23].

Cotton (*Gossypium* spp.) provides over 50% of the fibersused by the textile industry [24]. In the past decades, many breeding programs have worked to improve cotton resistance/tolerance to pathogens and insects, including transgenic Bt cotton [25]. PAs are known as a group of natural products that can protect plants against pathogens and insects [26]. Several studies reported cotton tissues produce PAs [27,28,29], but molecular breeding for PA-rich cotton cultivars still faces many obstacles blocks because the PA biosynthesis pathway in cotton remains unclear. In our previous studies, we characterized an anthocyanidin reductase (ANR) from upland cotton fiber, a key enzyme involved in PAs’ later biosynthesis steps [29]. To date, the steps of PAs biosynthesis before ANR in cotton need further to be elucidated.

We previously demonstrated that upland cotton produces PAs in many tissues in characterizing an ANR [29]. The evidence shows that *DFR* could be expressed in cotton, but it has not been characterized yet. In this study, we isolated a cDNA encoding a DFR isoenzyme from the fiber of upland cotton (designated as *GhDFR1*), and revealed the function of GhDFR1 by virus-induced gene silencing (VIGS) in cotton and recovering the phenotype of the *TT3* mutant in *Arabidopsis.*

## 2. Results and Discussion

### 2.1. Cloning of DFR Gene from Fiber of G. hirsutum

**Figure 2:**
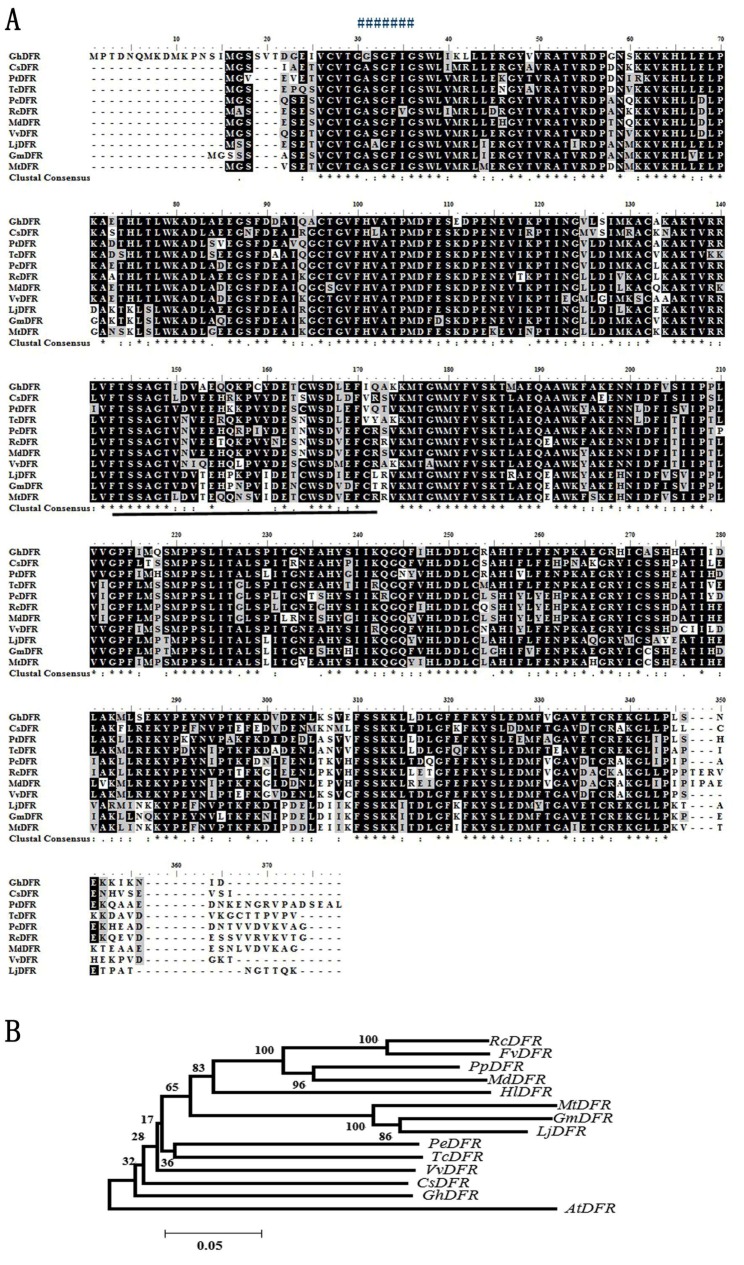
A sequence alignment (**A**) and a phylogenetic tree (**B**) obtained from deduced amino acid sequences of DFR homologs. (**A**) An amino acid sequence alignment was obtained from 10 deduced DFR amino acid sequences that were aligned on NCBI; (**B**) An unrooted phylogenetic tree obtained from 14 sequences. “*”: the same amino acid in all 10 sequences; “:”: conserved amino acid residues; “.”: half conserved amino acid residues; “#######”: Rossmann domain GXXGXXG; the underline showed potential domain involved in DFR substrates specificity.

Previously we characterized a cotton ANR protein which comes after DFR in the PA biosynthesis pathway. Here, we isolated a DFR cDNA from cotton fiber. Based on the available EST sequence of *G. hirsutum*, a pair of specific primers was designed to amplify and clone *GhDFR1* from early development 9 DPA fiber. As a result, an approximately 1 kb in size cDNA fragment has been isolated by reverse transcript PCR. This cDNA fragment was subsequently cloned into a T-easy vector for sequencing. After sequencing, the sequence was composed 1068 nucleotides including start and stop codons which encodes 355 amino acids. The deduced amino acid sequence was analyzed by a blast search at NCBI. The results of alignment showed the amino acid sequence of GhDFR1 is similar to *Citrus sinensis* DFR (CsDFR) [30], possessing a high conservation at the glycine-rich Rossmann NADPH/NADH-binding domain, which is composed of G-X-X-G-F-X-G. In addition, amino acids from 143 to 172 were shown to be relevant for determining substrate specificity (Figure 2A). The phylogenetic analysis revealed that GhDFR1 and CsDFR were in the same clade (Figure 2B).

**Figure 3:**
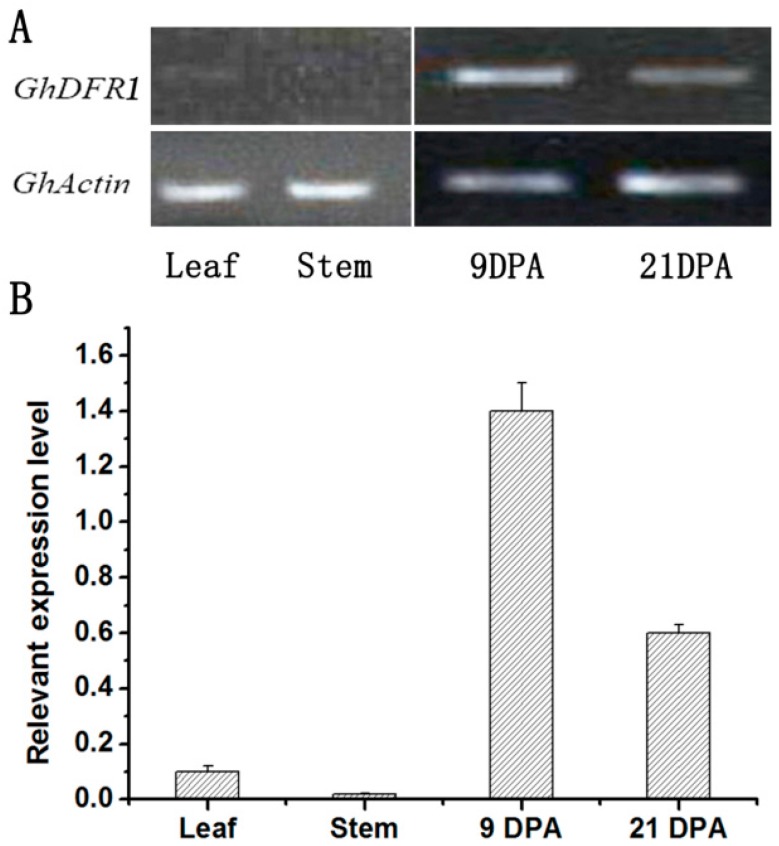
*GhDFR1* expression pattern in cotton. (**A**,**B**): Semi-RT-PCR (**A**) and qPCR (**B**) results show expression levels of *GhDFR1* at stem and leaf tissues, and two points developing fiber in *G. hirsutum*. DPA: day after flower fiber; *Actin*: β-actin as reference gene.

Semi-RT-PCR and quantitative real-time PCR (qPCR) have been performed to analyze the *GhDFR1* expression profile in cotton. As shown in Figure 3A,B, *GhDFR1* expressed in vascular tissues (stem and leaf) and both early (9DPA) and later (21 DPA) development fibers, but the expression level in vascular tissue is very low compared to fibers (Figure 3).

### 2.2. Silencing GhDFR1 Affected Flower Coloration and Content of Anthocyanins in Cotton Plants by VIGS

**Figure 4:**
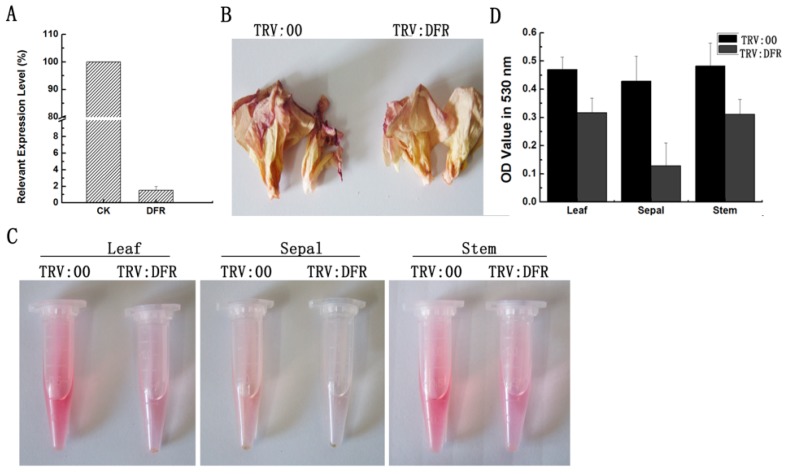
Tissue phenotypes after *GhDFR1* knockdown in *G. hirsutum* by VIGS. (**A**) *GhDFR1* expression levels in *GhDFR1*-silenced plants and the control by qPCR; (**B**) Petal phenotypes of *GhDFR1*-silenced plants and the control; (**C**) Comparison of red color from anthocyanin extracts from leaf, stem, and sepal of *GhDFR1*-silenced plants and the control; (**D**) Absorbance values of anthocyanin extracts from leaves, stems, and sepal of *GhDFR1*-silenced plants and the control.

According to the VIGS system in *G. hirsutum* and *G. barbadense* that we recently developed [29,31], in this study, we silenced the *GhDFR1* expression in cotton for analyzing its function. Partial fragments of *GhDFR1* open reading frames were subcloned to generate a pYL156-*GhDFR1* recombinant vector, which was then introduced into 10-day-old plants mediated by *Agrobacterium tumefaciens* GV 3101 strain. The *GhDFR1*-silenced plants and the control plants injected with *A. tumefaciens* GV 3101 containing empty vector pYL156 grew in the greenhouse until flowering. To investigate the silence efficiency of *GhDFR1* in *GhDFR1*-silenced plants, qPCR was firstly employed to analyze the expression levels of this gene. As shown in Figure 4A, the *GhDFR1* expression levels in *GhDFR1*-silenced plants were almost 50-folds lower than the control. The flowers of *GhDFR1*-silenced plants showed reduced reddish petal of 4 DPA compared to the control (Figure 4B). These results indicated that GhDFR1 affected anthocyanin biosynthesis in flowers of *GhDFR1*-silenced plants. To further investigate the decreased levels of anthocyanins in leaves, stems, and sepals, we extracted anthocyanins from these tissues using 50% acidic methanol. After removal of chlorophyll and other pigments by chloroform, the anthocyanin extracts from these three tissues of the control clearly showed a redder color than *GhDFR1*-silenced plants (Figure 4C). According to the absorbance at 530 nm measured by spectrophotometer, the OD values of leaf, stem, and sepal anthocyanin extracts of *GhDFR1*-silenced plants are significantly lower than those of the control (Figure 4D).

### 2.3. Silencing GhDFR1 Affected Structure and Content of PAs and Flavan-3-ols in Cotton Plants by VIGS

**Figure 5:**
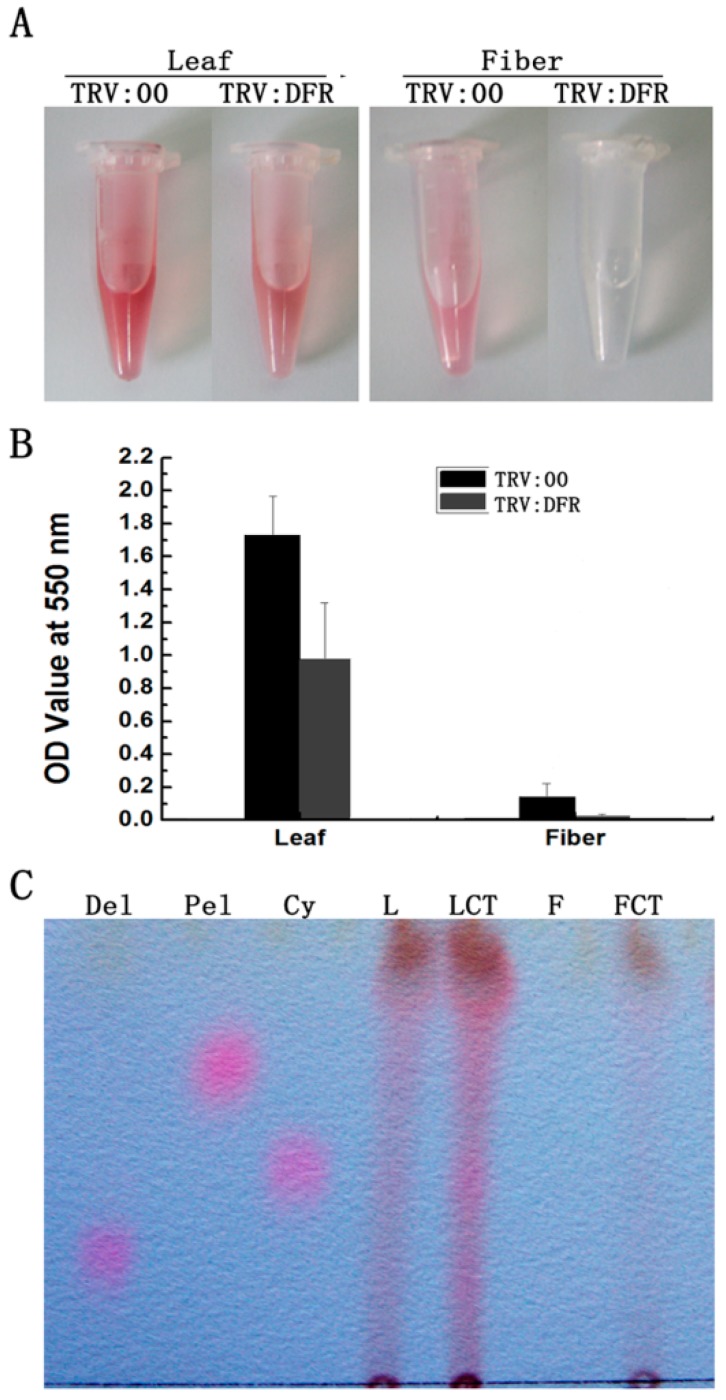
Comparison of PAs from *GhDFR1*-silenced plants and the control. (**A**) Color intensity derived from cleavage due to the butanol:HCl boiling of PAs; (**B**) Absorbance values of anthocyanidins resulting from PA cleavage at 550 nm; (**C**) TLC profiles showing anthocyanidins intensities from butanol:HCl cleaved PAs; Cy: cyanidin; Pel: pelargonidin; Del: delphinidin.

To investigate if silencing *GhDFR1* affecting PA biosynthesis, PAs and flavan-3-ols from fibers and leaves of *GhDFR1*-silenced plants and the control were extracted. The extractions from leaves and fibers hydrolyzed by boiling butanol:HCl produced a reddish coloration, which resulted from PA-derived anthocynidins [32]. The intensity of reddish coloration of extractions from *GhDFR1*-silenced plant was lighter than the control (Figure 5A). Further absorbance measurements at 550 nm showed the OD values were significantly lower in extractions from *GhDFR1*-silenced plant tissues than from the control (Figure 5B). Compared to leaves, the OD values decreased more significantly in fibers.

**Figure 6:**
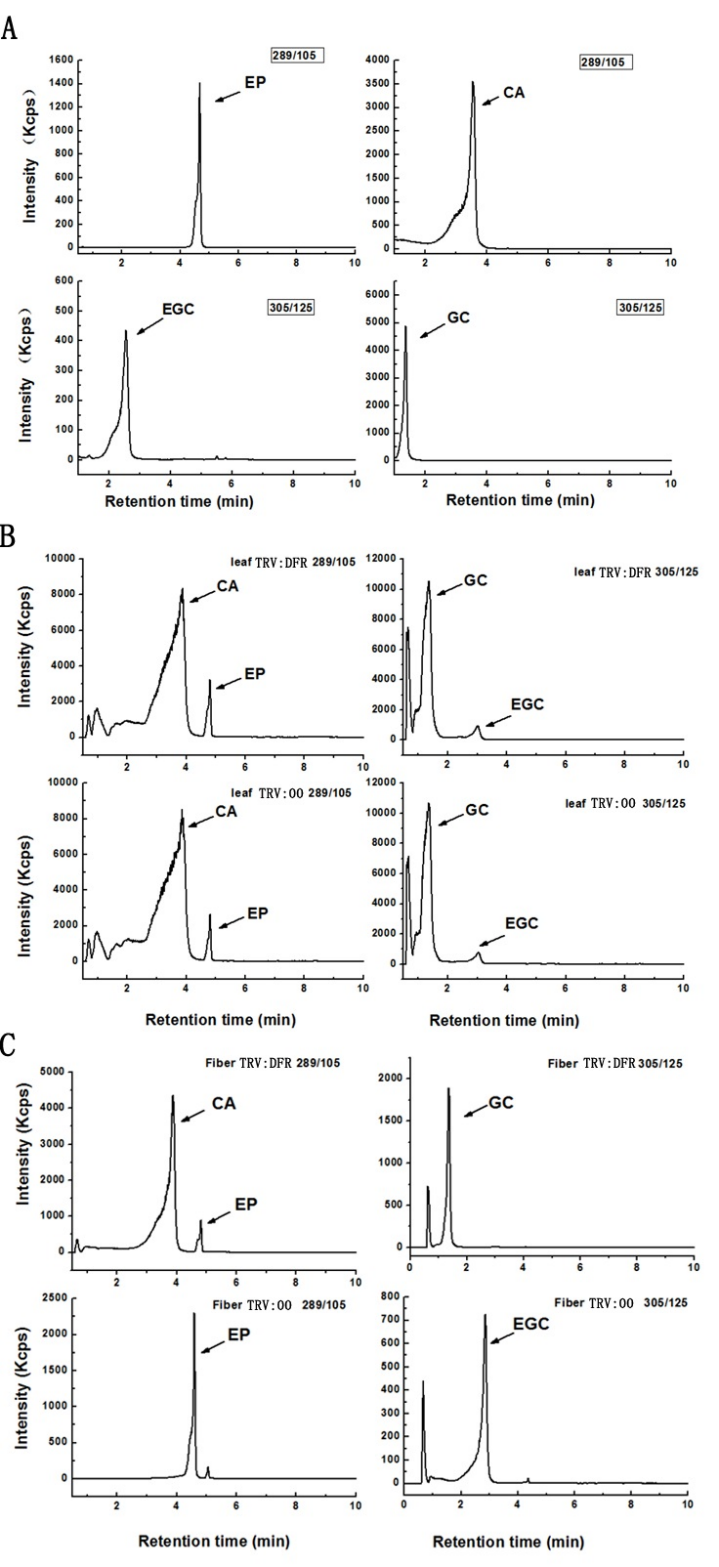
LC-MS-based profiling determines the flavan-3-ols from leaves and fibers of *GhDFR1-*silenced plants and the control in MRM mode. (**A**) Authentic standards of four flavan-3-ols in in *m/z* 305→125 [M − H]^−^ transition and *m/z* 289→109 [M − H]^−^ transition; (**B**,**C**) flavan-3-ols from leaves (**B**) and fibers (**C**) of *GhDFR1*-silenced plants and the control. CA: (–)-catechin; EP: (–)-epicatechin; GC: (–)-gallocatechin; EGC: (–)-epigallocatechin.

Based on further analysis of the boiled extracts by TLC profiling, the results showed cyanidin, delphinidin, and pelargonidin produced from butanol:HCl boiling of PA extracts from *GhDFR1*-silenced plant tissues show remarkably lower coloration intensity compared to the control, indicating that the PA contents of these tissues were lower in *GhDFR1*-silenced plants than those in the control. Especially, fiber PA-derived anthocynidins were not observed in TLC profiling (Figure 5C). The structures and contents of flavan-3-ols (monomers of PAs, also polyphenols) were estimated by LC-MS analysis. The resulting data showed that four flavan-3-ols including (–)-catechin, (–)-epicatechin, (–)-gallocatechin, and (–)-epigallocatechin could be detected by LC-MS in leaf tissues of both *GhDFR1*-silenced plants and the control. However, the contents of these four flavan-3-ols did not differ significantly between *GhDFR1*-silenced plants and the control, possibly due to low expression level of *GhDFR1* in leaf (Figure 6A,B). Interestingly, (–)-epicatechin and (–)-epigallocatechin had been detected in fiber of the control, but in *GhDFR1*-silenced plants, (–)-catechin, (–)-epicatechin, and (–)-gallocatechin were measured. (–)-Epigallocatechin was only present in the control, (–)-catechin and (–)-gallocatechin were only detected in *GhDFR1*-silenced plants. Of these flavan-3-ols, (–)-epicatechin contents of fiber of *GhDFR1*-silenced plants decreased significantly compared to the control (Figure 6C).

### 2.4. Overexpression of GhDFR1 Gene in Arabidopsis thaliana TT3 Mutant

**Figure 7:**
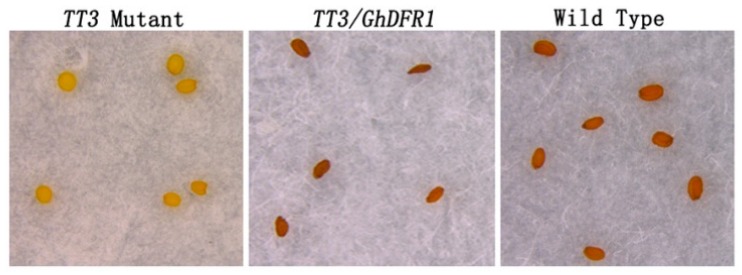
Ectopic expression of *GhDFR1* in an *Arabidopsis thaliana TT3* mutant. Seed coloration of *TT3* mutant, ectopically expression *GhDFR1*, and Columbia wild-type plants.

The *A. thaliana TT3* mutant is characterized by knockout of *DFR*, leading to a lack of flavan-3-ols and PAs in the seed coat, which consequently shows a white coloration, lacking the brown which results from oxidation of PAs and flavan-3-ols [6]. To investigate *GhDFR1* function in plants, it was ectopically expressed in *A. thaliana TT3* mutant. Using of a flower dip transformation method, transgenic *GhDFR1* plants were obtained. The coloration of T1 *GhDFR1* transgenic seeds showed deep brown coloration similar to that of wild type seeds (Figure 7). This result indicated the overexpression of *GhDFR1* in *TT3* mutant reconstructed the mutated PAs biosynthesis pathway.

### 2.5. Discussion

DFR is a key enzyme to control the biosynthesis of at least two groups of flavonoids—anthocyanins and PAs—which provide plant pigments and resistance/tolerance to many biotic and abiotic stresses, respectively. In the past decades, many studies have focused on DFR’s functions due to its contribution to the coloration of flowers [1,2,7,8,9]. Some reports showed that DFR was involved in the biosynthesis of PAs participating in plant responses to abiotic and biotic stresses [10,33]. For instance, *DFR* gene was strongly induced in *Populus tremuloides* damaged by insect herbivores or treated with methyl jasmonate, leading to an accumulation of PAs [10]. DFRs from *Brassica rapa* showed responses to cold and freezing stress treatments [33]. Especially, PAs had been shown to limit the pest growth due to nutrition indigestion [34,35]. In cotton, a major goal of breeding is resistance/tolerance to pests to decrease the tremendous yield losses caused by pests [24]. Therefore, breeding of PAs-rich cultivars should be a considerable strategy through some candidate genes including *GhDFR1*.

In this study, knock-down of *GhDFR1* expression in *GhDFR1*-silenced plants, only 2% of the control (Figure 4A), led to decreased anthocyanins in leaves, sepals, stems and flowers. Analysis of butanol:HCl boiling of methanol extracts indicated that the PA monomers from fibers and leave tissues decreased in *GhDFR1*-silenced plants. In addition, the TLC profile of PA-derived anthocyanidins showed three anthocyanidins are reduced in *GhDFR1*-silenced plants, which demonstrates GhDFR1 can accept three substrates. PAs biosynthesis was reconstructed in an *A. thaliana TT3* mutant by the ectopic expression of *GhDFR*. Taken together, the results demonstrate that the *GhDFR1* makes a great contribution to the biosyntheses of PAs and anthocyanins in cotton.

In this present study, according to an LC-MS assay, the flavan-3-ols showed similar contents in leaves between *GhDFR1*-silenced plants and the control, probably due to the low expression level of *GhDFR1* in leaves. Interestingly, in fibers, the flavan-3-ols showed different structures and concentrations. (–)-Epicgallocatechin was not present in *GhDFR1*-silenced plants, but the contents of (–)-catechin and (–)-gallocatechin were increased significantly compared with the control. However, this metabolic shift of flavan-3-ol structures still need to be further investigated.

## 3. Experimental Section

### 3.1. Plant Materials

Seeds of *G. hirsutum* cultivar TM-1 were germinated and grown in a greenhouse. After two week growth at 25 °C and 16/8 h light/dark, seedlings from cotyledon expansion to 4th true leaf emergence were used for VIGS assay. After infection, the *GhDFR1*-silenced plants were growth in a greenhouse under the same conditions described before. After flowering, the developing fiber, leaves, stems, petals, and sepal were collected for further semi-RT-PCR, qPCR, PA, and anthocyanin assays.

### 3.2. Total RNA Extract, Semi-RT-PCR, and qPCR

Total RNA of different tissues were extracted by using Plant Total RNA Isolation Kits (Sangon, Shanghai, China). One microgram DNA-free RNA from *G. hirsutum* young leaf, stem, and developing fiber were reverse transcribed by using BluePrint 1st strand cDNA Synthesis Kit (Takara, Dalian, China) following the manufacturer’s protocol. Semi-RT-PCR was controlled by PCR using 1 μL cDNA in a 25 μL reaction with *beta-Actin* primers. In this study, 25 cycles of PCR amplification were performed for *beta-Actin* and 30 cycles were performed for the *GhDFR1*. PCR products were analyzed by 1% agarose electrophoresis. A pair of primers, the forward primer 5′-CAGATTTAGCTGAAGAGGGAAG-3′, and the reverse primer 5′-GCTCTGCCATTGTCTTGGAG-3′, was designed based on the *G. hirsutum DFR* gene EST sequence. The qPCR was performed with the SYBR Premix Ex Taq kit (Takara) according to the manufacturer’s guidelines. All PCR were run with three technical replicates and three biological replicates on an IQ5 multicolor detection system (Bio-Rad, Hercules, CA, USA). *GhDFR1* gene expression was calculated using the ddCt algorithm. To normalize gene expression, *beta-Actin* was used as an internal standard. The specific primers of *GhDFR1* and *Actin* genes are designed to use qPCR analysis, which are the primer pairs of *GhDFR1* (5′-TGCCAACTGATAATCAAATG-3′ and 5′-TGAGCCATGAACCAATGAAC-3′) and *Actin* (5′-TTACTCAGTATTGTAAAAGATGGCC-3′ and 5′-AGCATCATCACCAGCAAAAC-3′).

### 3.3. Cloning of GhDFR1 from G. hirsutum

A pair of primers for clone *GhDFR1* complete open reading frame, the forward primer 5′-ATGCCAACTGATAATCAAATG-3′ containing start codon, and the reverse primer 5′-TCAGTCTATGTTTTTGATCT-3′ containing stop codon, was designed based on *G. hirsutum DFR* gene EST sequence. One microgram DNA-free RNA from *G. hirsutum* young fiber tissue were reverse transcribed by using BluePrint 1st strand cDNA Synthesis Kit (Takara) following the manufacturer’s protocol. The follow PCR thermo cycles was carried out using *Ex-taq* DNA polymerase (Takara) at 94 °C for 5 min, 30 cycles of 94 °C for 30 s, 60 °C for 30 s, and 72 °C for 45 s; followed by a 10 min extension at 72 °C. After gel purification, the PCR products were subcloned into a T-easy vector (Promega, Madison, WI, USA) for sequencing.

### 3.4. VIGS Mediated by Agrobacterium

Seeds of *G. hirsutum* cultivar TM-1 were germinated and grown in the greenhouse. Seedlings from cotyledon expansion to 4th true leaf emergence were used for VIGS assay. The pYL156-DFR1 was constructed as described before [31]. The pTRV-RNA1, pYL-156-DFR1, and pYL-156 (empty vector) were transformed into *A. tumefaciens* strains GV3101 by electroporation, respectively.

Agrobacteria were grown overnight at 28 °C, 180 rpm in LB medium containing 50 μg/mL kanamycin, 25 μg/mL gentamicin, 10 mM MES, and 20 μM acetosyringone. The cells were collected by 4000 rpm centrifugation at room temperature for 10 min, and resuspended in MMA (10 mM MES, 10 mM MgCl_2_, 200 μM acetosyringone) solution to a final OD_600_ of 1.5. The suspensions were incubated at room temperature for 3 h. Agrobacteria containing pTRV-RNA1 and pYL156-DFR1 or pYL-156 were mixed at ratio 1:1, and infiltrated into fully expanded cotyledons by using needleless syringe.

### 3.5. Extraction and Assay of Anthocyanins

One hundred mg of fresh material was ground to a fine powder in liquid nitrogen, which was transferred into 1.5 mL tube. 0.5% HCl in 50% methanol (1 mL) was added and allowed to incubate in room temperature for 30 min. The tubes were centrifuged at 10,000 rpm for 10 min, and the liquid phase was transferred into a new tube. Chloroform (0.2 mL) was added in tube, which was vortexed for 30 s and shortly centrifuged to remove the chloroform phase. This step was repeated twice to remove the chlorophyll completely. The concentration of anthocyanins in rough extracts was measured by recording absorbance at 530 nm by UV-spectrum.

### 3.6. Extraction of the Soluble PAs

All of steps were performed in room temperature. One hundred mg of fresh material was ground to a fine powder by liquid nitrogen and transferred into a 1.5 mL tube. Acetone (1 mL) was added into the 1.5 mL tube immediately. Tubes were vortexed for 30 s, and centrifuged at 6000 rpm for 3 min, and acetone phase was transferred into a new 5 mL tube. After repeating this sequence twice, the acetone phases were combined, followed by vacuum drying to completely remove the acetone. To the residue milliQ water (500 μL) and chloroform (200 μL) were added, and tube was vortexed for 30 s, then briefly centrifuged to remove the chloroform phase. This step was repeated twice to remove chlorophyll completely. Ethyl acetate (500 μL) was added to the water phase, the tube was vortexed for 30 s and briefly centrifuged, and then the ethyl acetate phase was transferred to a new tube. After repeating twice the ethyl acetate phases were combined and vacuum dried to remove the ethyl acetate. The residue was dissolved in 100 μL methanol.

### 3.7. PAs Hydrolysis and TLC Assay

Each 50 µL PAs extraction was mixed with 950 µL butanol:HCl (95:5, *v*/*v*) and the mixture was then boiled for 1 h. After cooling to approximately room temperature, A 550 nm OD value of each sample was measured by spectrophotometry (Shimadzu, Tokyo, Japan). After the measurement, samples dried by rotary evaporator and then resuspended in 50 µL 0.1% HCl methanol. Ten µL were loaded for the TLC assays. The TLC plates used were cellulose F-200 µM chromatography layer. The eluting solvent system consisted of hydrochloric acid–acetic acid–water (3:30:10, *v*/*v*). Authentic standards of pelargonidin chloride, cyanidin chloride and delphinidin chloride (Sigma, St. Louis, MO, USA) were loaded as references.

### 3.8. LC-MS Analysis of Flavan-3-ols

The LC-MS analysis was carried out on an AB SCIEX QTRAP 4500 system (AB SCIEX, Foster, CA, USA). In brief, metabolites were separated on an Eetend-C18 (2.1 mm × 100 mm, 3.5 μm) reverse phase column. The elution solvent system composed of 0.1% formic acid (solvent A) and acetonitrile (solvent B). A gradient program composed of different ratios of solvent A to solvent B was developed to elute metabolites. The program consisted of 95:5 to 0:100 (0–12 min), 0:100 (12–18 min), 0:100 to 95:5 (18–19 min), 95:5 (19–24 min). The injection volume of methanol extraction was set at 5 μL. The flow rate was set at 400 μL/min. The authentic standards (–)-catechin, (–)-epicatechin, (–)-gallocatechin, and (–)-epigallocatechin (Sigma) were injected as quality control. Four potential flavan-3-ols were detected at 280 nm. The separated metabolites were followed by MS analyses conducted in negative-ion mode. The operating parameters were optimized as follows: collision gas (CUR): 20.0; collision gas (CAD): medium; IonSpray voltage (IS): −4500.0; temperature: 500.0 °C; ion source gas 1 (GS1): 55.0; ion source gas 2 (GS2): 55.0; declustering potential (DP): −90.0; entrance potential (EP): −10.0; collision energy (CE): −30.0; collision cell exit potential (CXE): −18.0. Ion detection was performed in multiple reaction monitoring (MRM) mode where in *m/z* 305→125 [M − H]^−^ transition for (–)-gallocatechin and (–)-epigallocatechin and *m/z* 289→109 [M − H]^−^ transition for (–)-catechin and (–)-epicatechin.

### 3.9. Overexpression of GhDFR1 in A. thaliana TT3 Mutant

To further characterize the function of *GhDFR1*, we cloned its ORF immediately downstream of the 35S promoter in the T-DNA of pBI121 binary vector by replacing the *GUS* reporter gene. The recombinant vector pBI121-GhDFR1 was introduced into *A. tumefaciens* strain EHA 105 by electroporation using a micropulser apparatus (411BR 0454, Bio-Rad). The *A. thaliana TT3* mutant was purchased from SALK (SALK ID: CS2114). Seed germination and seeding growth followed the standard *Arabidopsis* growth protocol. Genetic transformation followed the floral dipping transformation protocol [36]. The growth of infected plants and seed harvesting followed the same protocol as previous reported [29,32]. Under the standard growth conditions, T1 plants begun to develop flowers about 60 days after seed germination. Wild type (Columbia ectype) and *TT3* mutant plants were grown as controls. The mature seeds were collected for photographs.
